# Modulation of Immune Function by Polyphenols: Possible Contribution of Epigenetic Factors

**DOI:** 10.3390/nu5072314

**Published:** 2013-06-28

**Authors:** Alejandro Cuevas, Nicolás Saavedra, Luis A. Salazar, Dulcineia S. P. Abdalla

**Affiliations:** 1 Center of Molecular Biology and Pharmacogenetics, Scientific and Technological Bioresource Nucleus, Universidad de La Frontera (BIOREN-UFRO), Temuco 4811230, Chile; E-Mails: acuevas@ufro.cl (A.C.); nsaavedra@ufro.cl (N.S.); luis.salazar@ufrontera.cl (L.A.S.); 2 Department of Clinical and Toxicology Analysis, Faculty of Pharmaceutical Sciences, University of Sao Paulo, Sao Paulo 05508-900, Brazil

**Keywords:** polyphenols, immune system, epigenetic

## Abstract

Several biological activities have been described for polyphenolic compounds, including a modulator effect on the immune system. The effects of these biologically active compounds on the immune system are associated to processes as differentiation and activation of immune cells. Among the mechanisms associated to immune regulation are epigenetic modifications as DNA methylation of regulatory sequences, histone modifications and posttranscriptional repression by microRNAs that influences the gene expression of key players involved in the immune response. Considering that polyphenols are able to regulate the immune function and has been also demonstrated an effect on epigenetic mechanisms, it is possible to hypothesize that there exists a mediator role of epigenetic mechanisms in the modulation of the immune response by polyphenols.

## 1. Introduction

Polyphenols are bioactive compounds characterized by the presence of large multiples of phenol structural units. These compounds are frequently found in beverages, fruits and vegetables, in which they provided the color and flavor, while also contributing in responses to UV radiation, pathogens and other damage in plants [1]. Commonly are classified according to its chemical structures into non flavonoids as phenolic acids and phenolic amides or flavonoids, subdivided by their substituents into isoflavones, neoflavonoids, chalcones, flavones, flavonols, flavonones, flavononols, flavanols, proanthocyanidins and anthocyanidins [2]. Numerous studies have attributed to them a wide range of biological activities including anti-inflammatory [3], antioxidant [4], cardiovascular protective [5] and anti-cancer actions [6], among many others [7]. The evidence about the effects of polyphenols on immune function is abundant; however, the mechanisms involved in these actions are not fully understood. The biochemical effects of phytochemical compounds are involved in the mechanisms by which the polyphenols exert their function. The anti-oxidant action (ROS scavenging, oxidative stress protection, thiol-redox stabilization, membrane lipid peroxidation attenuation) or pro-oxidant activity (ROS production, thiol-redox alteration, membrane lipid peroxidation, oxidative stress) of polyphenols are able to regulate the epigenetic factors by oxidant and thiol-redox-mediated signaling modulation [8]. For instance, it was shown that 4-HNE, an α,β-unsaturated hydroxyalkenal produced by lipid peroxidation, can induce allosteric inhibition of SIRT3 activity by covalent modification at Cys^280^, resulting in an altered mitochondrial protein acetylation [9]. In this sense, some polyphenols can act as scavenger of α,β-unsaturated aldehydes and thus inhibit or restrain carbonyl stress-associated diseases [10,11].

**Figure 1:**
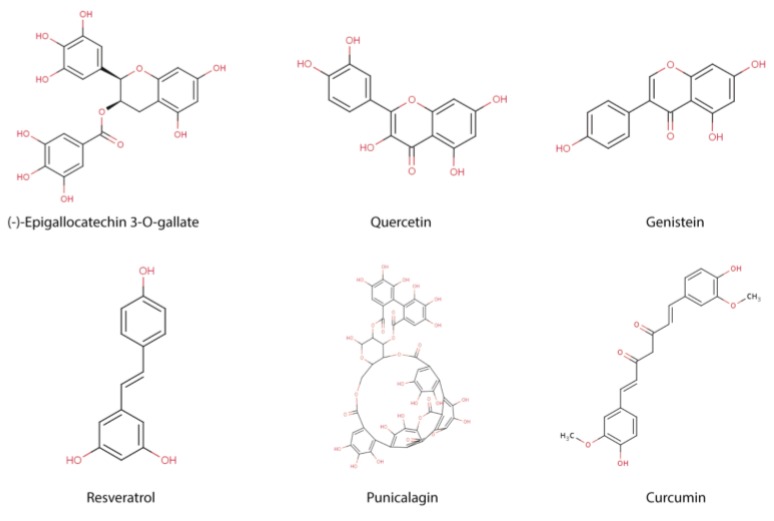
Chemical structures of different polyphenols involved in the modulation of epigenetic mechanism.

Polyphenols of several sources have shown a modulator effect on epigenetic mechanisms as DNA methylation, histone modifications and posttranscriptional regulation by microRNAs (Figure 1), and these mechanisms in turn, can modulate the immune system influencing both activation and differentiation of multiple cell types involved in the immune response. However, poor evidence has been described about the epigenetic contribution on modulation of immune system by polyphenols. In this review, we summarized the immune factors modulated by polyphenols, as well as the immune modulation by epigenetic factors and the possible relation between polyphenols and modulation of immune system through epigenetic mechanisms.

## 2. Immune Function and Polyphenols

Many studies support the immune modulation actions by polyphenols from diverse sources, describing immune modulator effects on different populations of immune cells [12,13]. In mice receiving oral treatment with polyphenols rich extracts from date palm tree, an increment of the immunocompetent cells, incluinding T helper 1 (Th1), natural killer (NK), macrophages and dendritic cells (DCs) in both Peyer’s patches and spleen [12] was observed. Similar effects were obtained in response to treatment of aged rats with polyphenols from *Cassia auriculata*, increasing splenic T and B cells [13]. Other studies have been reported mechanisms associated to specific cell groups among the different immune cells modulated by polyphenols.

Regulatory T cells (T_reg_) constitute a T lymphocyte subset which plays an important role in immunity tolerance and autoimmunity control, being its dysfunctional activity associated to the development of diseases, such as allergy [14], inflammatory bowel disease [15], multiple sclerosis [16], rheumatoid arthritis [17] and type I diabetes [18]. These cells are originated principally in the thymus and can be differentiated from naïve CD4^+^ T cells in a process under transforming growth factor beta (TGF-β) stimulus, which leads to the transcription factor forkhead box P3 (Foxp3) expression, giving to T_reg_ its characteristic phenotype (CD4^+^CD25^+^Foxp3^+^) [19]. Due to the important function of this cellular group in the control of immune function, several studies have been conducted to explore its modulation using compounds with biological activity. In this line, it has been described the effect of Epigallocatechin-3-gallate (EGCG), a member of the flavanols class found in green tea. *In vitro* experiments, using Jurkat T cells treated with EGCG (2 or 10 μM) or green te at 50 uM, achieved an increment in Foxp3 and IL10 gene expression. In parallel, a group of Balb/c mice was injected daily with EGCG 50 mg/kg for seven days, increasing T_reg_ frequency and number in spleens, pancreatic lymph nodes, and mesentheric lymph nodes. Moreover, T_reg_ cells obtained from treated group were able to suppress T cell functions, showing a reduction on T cell proliferative capacity and interferon gamma (IFNγ) production, indicating the functionality of the stimulated cells [20]. Similarly, assays conducted to evaluate the effect of Baicalin, a flavone isolated from the Chinese medicinal herb Huangqin, demonstrate in HEK 293 T cells an increase of Foxp3 gene expression similar to that exhibited under TGF-β stimulus as well as an induction of functional T_reg_ from splenic CD4^+^CD25^−^ T cells [21]. Recently, a possible mechanism of Foxp3 induction by flavonoids was described. Assays were conducted to evaluate the capacity of certain flavonoids to act as agonists of aryl hydrocarbon receptor (AhR), a transcription factor capable of binding xenobiotic-responsive elements (XRE) in promoter regions of certain genes, among which Foxp3 is included. Naringenin showed AhR ligand activity and achieved an important suppression of responder CD4^+^ T cells in either presence or absence of TGF-β [22].

T helper (Th) cells are also involved in the control of autoimmunity and are associated with induction of the autoimmune process. As T_reg_, Th cells are originated from naïve CD4^+^ T cells differentiated into three types of them: Th1, Th9 and Th17 tcells. These cell subtypes are originated in according to specific stimulus that leads to the activation of characteristics transcription factors, being considered masters regulators the T-bet, RORγt and PU.1 for Th1, Th17 and Th9 respectively [23]. Using an animal model of experimental autoimmune encephalomyelitis, the effect of the diet supplementation with EGCG was evaluated, showing a decrease on Th1 and Th17 populations, together with lower expression of T-bet and RORγt. These findings are correlated with data later obtained by the same group, describing an inhibitory effect on STAT1 and STAT4 phosphorylation, signal transducers involved in the Th1 differentiation. Furthermore, EGCG showed a reduction of Th9 cells together with decreased expression of the PU.1 [24]. Baicalin treatment also has an effect on T helper cells differentiation. *In vitro* assays using naïve T cells from C57BL/6 mice under Th17 differentiation conditions and Baicalin treatment showed a decreased expression of interleukin 17 (IL-17), cytokine produced characteristically by these cells. Moreover, this finding was described together with a reduction of RORγt expression [25].

Other important population of lymphocytes is that constituted by B cells that are characterized by the expression of B cell receptor (BCR). These cells are involved in humoral immunity and have the capacity of express diverse immunoglobulin (Ig). Moreover, these cells play a role in the regulation of immune homeostasis independently of Ig production as CD4^+^ T cells activation, regulation of dendritic cells and tumor immunity [26]. The effects of polyphenols on immune activity of these cells have been poorly described, although some evidence has been shown. Using peripheral blood mononuclear cells obtained from healthy adult individuals stimulated by pokeweed mitogen (PWM), acting as inductor of proliferation and Ig synthesis in B lymphocytes, an increment on proliferation of CD19^+^ cells was observed with resveratrol without changes of Igs production [27]. This is in contrast to a similar study, in which the treatment with polyphenolic fraction purified from cacao liquor inhibited the proliferation of CD19^+^ cells and IgG production [28]. The treatment of U266 cells with Green tea, rich in EGCG content, showed a relevant effect on the IgE production exhibiting a dose- and time-dependent decrease of this immunoglobulin [29], demonstrating that polyphenols are also relevant in the modulation of B cell function.

The most studied activity of polyphenols on immune function is related with modulation of inflammatory response in macrophages. These cells plays a key role in the initiation of inflammatory response mainly by the production of pro-inflammatory mediators as prostaglandin E2 (PGE_2_) and cytokines as interleukin 6 (IL-6) and tumor necrosis factor-alpha (TNF-α). Sometimes, the immune response becomes persistent promoting the development of chronic pathologies as atherosclerosis, rheumatoid arthritis and inflammatory bowel disease among other immune-related diseases [30]. Thus, the search for immune modulators has been an important focus of study, emphasizing the role of polyphenols as an interesting alternative. In this regard, *in vivo* and *in vitro* studies have demonstrate that one of the main effects of polyphenols on macrophages is the inhibition of keys regulators of inflammatory response, being the repression of ciclooxygenase-2 (COX-2), inducible nitric oxide synthase (iNOS) and the cytokines TNF-α, interleukine-1-beta (IL-1-β) and IL-6 among the most consistent effect [31]. In murine RAW 264.7 macrophages stimulated by lipopolysaccharide (LPS), a dose dependent decrease on iNOS, IL-1-β and IL-6 mRNA expression and the subsequently nitric oxide (NO), IL-1-β and IL-6 production was observed, when the cells were pre-treated during 1 h with Chinese propolis [32]. Other studies also showed inhibitory effects on iNOS, COX-2 and the inflammatory cytokines TNF-α, IL-1-β e IL-6 after treatment with an crude extract of *Lonicera japónica* Thunb (*Caprifoliaceae*) [33] or *Kalanchoe gracilis* [34]. The same inhibitory effect on cytokines was induced with 7-*O*-methylnaringenina, associating to these changes a decreased phosphorylation of extracellular signal-regulated protein kinases 1 and 2 (ERK1/2) and c-Jun *N*-terminal kinases (JNK), both important factors involved on the lipopolysaccharide (LPS) activation of cytokines [35].

The effect of analog polyphenolic compounds or compounds exhibiting structural modifications has also been evaluated. The curcumin analog EF31, inhibited the expression and secretion of TNF-α, IL-1-β and IL-6 [36]. Similarly, RVSA40 an analog of resveratrol, showed an inhibition of TNF-α and IL-6, together with up-regulation of anti-inflammatory cytokine interleukin-1-alpha (IL-1α). Furthermore, RVSA40 inhibited the activity of the transcription factor STAT3, constitutively expressed in HEK293 cells, promoting its dephosphorylation by a protein tyrosine phosphatase mediated effect [37]. Studies conducted in macrophages derived from THP-1 monocytes, demonstrated similar effects to these previously described [34,35,36]. Extracts of chamomile, meadowsweet, willow bark and isolated polyphenols present in these extracts as quercetin, apigenin and salicylic acid, were able to suppress the secretion of TNF-α and IL-6 without IL-1β modulation in THP-1 monocytes differentiated with *phorbol*-12-myristate-13-acetate (PMA) [38]. Inhibitory effects on TNF-α and Interleukin 8 (IL-8) were observed after treatment with an extract of *Cydonia oblonga* in THP-1 stimuled with LPS. In these conditions, the phosphorylation of p38 and Akt was also reprised together with an increment of interleukin 10 (IL-10) and IL-6 secretion. The inhibitory effect on TNF-α was partially mediated by the increased IL-6 production acting as a negative feedback and thus limiting the acute inflammatory response [39,40].

Oxidative stress is a well-known cause of persistent chronic inflammation due to its ability to active transcription factors such as NF-κB, AP-1, p53, HIF-1α, PPAR-γ, β-catenin/Wnt, and Nrf2 [41]. Considering its importance, the effect of polyphenols on oxidative stress has also been studied. The treatment of THP-1 cells PMA stimulated and treated with infusion of yerba mate (*Ilex paraguariensis*) or chlorogenic acid, its main polyphenolic constituent, can increase both mRNA expression and activity of the antioxidant enzyme paraoxonase 2 (PON-2). The same effect was observed in peripheral blood mononuclear cells of healthy women [42]. Similarly, the treatment of THP-1 and J774A.1 cells with punicalagin, the main polyphenol of pomegranate, prevents the loss of antioxidant activity of the plasma membrane induced by the exposition to acetylated low density lipoprotein (LDL) [43].

Respect to cell signaling implicated in these effects, some studies have been described the implication of MAPKs pathways. In bone marrow-derived macrophages, the flavonoid procyanidin C1 was able to decrease the secretion of TNF-α, IL-1β and IL-6, together with a repression of both COX-2 and TLR4 expression and p38 and ERK-1/2 phosphorylation [44]. The theaflavin, that inhibits the expression of Monocyte chemoattractant protein-1 (MCP-1), IL-6 and Intracellular adhesion molecule-1 (ICAM-1), also acts through blocking the MAPKs ERK1/2, JNK and p38 in bone marrow-derived macrophages from ICR mice [45]. These findings indicate that independently of treatment or the cellular model used, the modulation of TNF-α, IL-1β and IL-6 appears as common factor in the polyphenols modulatory effects together with MAPK dependent pathways.

These signaling pathways, together with IkappaB kinase (IKK)-NF-κB regulates the activity of important transcription factors as NF-κB (p50/p65) and AP-1 (c-Fos/c-Jun), which after activation can induce the expression of numerous genes encoding inflammatory mediators [46]. The treatment of macrophages with polyphenols modulate the phosphorylation of IkB kinases (IKKs) [36,40] and IκB alpha (IκBα) [32,33,35], reducing its degradation and promoting the retention of p65 subunit into the cytsol blocking subsequently the process of its translocation to the nucleus [33,44,45]. Moreover, in HEK293 cells, wich express NF-κB constitutively, a decreased activation of this transcription factor was observed [32]. This mechanism seems to be the most important one responsible for the effects of polyphenols in cellular models of inflammation.

Studies using *in vivo* models of inflammation have also shown anti-inflammatory effects on macrophages. In mice stimulated with LPS intraperitoneally injected, it was observed that the oral consumption of cinnamon aqueous extract induces a decrease in serum concentrations of TNF-α and IL-6. These factors were also suppressed in LPS stimulated-peritoneal macrophages, together with a lower activation of IκBα, p38, JNK y ERK 1/2 [47]. APOE knockout mice, fed a diet containing blueberries, also exhibited lower serum concentrations of TNF-α and IL-6, less expression of TNF-α in aortic tissue besides TNF-α and IL-6 inhibition in peritoneal macrophages stimulates with LPS or oxidized LDL. Furthermore, RAW 264.7 cells treated with the serum of these animals showed the same effects together with a decreased phosphorylation of IκBα, NF-κB p65 subunit, p38 and JNK [48]. Finally, in humans, a decreased inflammation score in patient with controlled consumption of bilberries was described, considering serum levels of IL-6, IL-12, as well as, high sensitivity C reactive protein (hsCRP) [49] and reduced ICAM-1, E-selectin, IL-6, CD40 antigen, CD40 ligand, IL-16, MCP-1 and vascular cellular adhesion molecule-1 (VCAM-1) in serum of patients with high cardiovascular risk with wine polyphenols consumption [50].

## 3. Immune Function Modulation by Epigenetic Mechanisms

Epigenetic mechanisms as DNA methylation, histone modification and microRNAs (miRNAs) are involved in the regulation of many biological processes through gene expression modulation at both transcriptional [51] and posttranscriptional levels [52].

DNA methylation regulates the gene expression at the transcriptional level, which is occurring mainly in the context of CpG dinucleotides forming clusters called CpG islands. Methylated CpG-islands are associated with gene silencing and can directly inhibit transcription by precluding the recruitment of DNA binding proteins from their target sites or promoting the recruitment of methyl-CpG-binding domain proteins, able of recruiting histone modifying- and chromatin-remodeling complexes over the methylated sequence [53]. Histones undergo posttranslational modifications also regulates gene expression with either inhibition or activation of transcription. Particularly, the tails of H3 and H4 histones can be covalently modified at several residues by different process including methylation, acetylation, phosphorylation and ubiquitination. Some of these modifications, such as histone lysine methylation, are known to recruit specific binding proteins, whereas acetylation at various residues have a structural role, making the nucleosome structure less or more accessible to transcription factors [54]. MiRNAs constitutes a posttranscriptional mechanism of gene expression regulation. They are short sequences of non-coding RNAs (21–25 nucleotides) and regulate the gene expression mainly by two mechanisms, transcriptional repression or mRNA cleavage [52].

**Figure 2:**
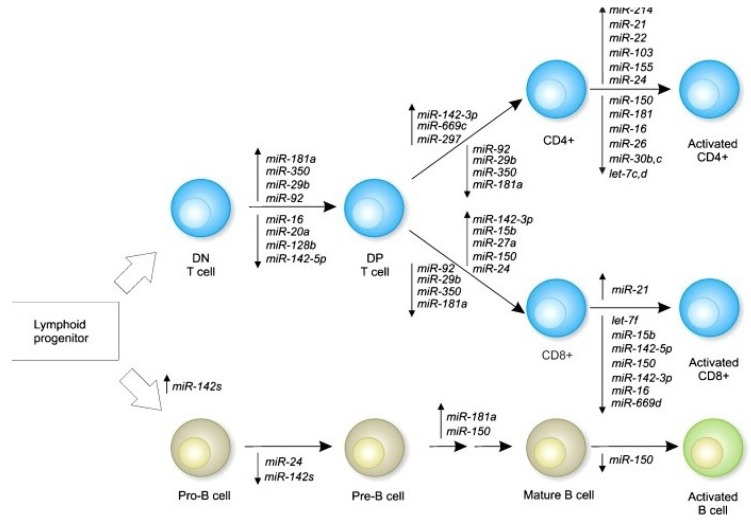
MicroRNAs involved in lymphoid differentiation. This figure summarizes the known changes in miRNA expression during differentiation/activation of T and B lymphocytes (Reprinted from [55] with permission from Elsevier).

During T- and B-cell differentiation, the influence of epigenetic mechanisms has been showed, particularly those mediated by microRNAs (miRNAs) that are dynamically regulated [55]. In this regard, miR-181a was identified as a differentially expressed miRNA in murine hematopoietic organs what suggested a role for this miRNA in the development of both T and B cells (Figure 2). Moreover, high expression of miR-181a induced an increase of B-lymphocyte *in vitro*, suggesting that miR-181a is a specific positive regulator for B-lymphocyte differentiation in mouse bone marrow. Additionally, overexpression of mir-181a produced two-fold reduction in the number of circulating T lymphocytes and about a 90% decrease in CD8^+^ subpopulation, demonstrating a role for this miRNA also in the development of T cells [56]. The higher miR-181a expression in mature T-cells was associated with an increased T-cell receptor (TCR) sensibility and its repression in immature T-cell decreasing the sensitivity to antigens [57]. This is consistent with the up-regulation of miR-181a in double positive thymocytes, and its down-regulation during thymocyte maturation [58]. Other associated miRNA is the miR-150, which was selectively expressed in mature resting T-cell but not in their progenitors or activated CD4^+^ and CD8^+^ T cells, showing higher expression in CD8^+^ cells compared with CD4^+^ cells [58,59]. The overexpression of miR-150 inhibited the transition from pro-B cell to the pre-B cell in mice transplanted with hematopoietic progenitor cells retrovirally transfected [60]. In addition, it was showed that lethally irradiated mice transplanted with hematopoietic progenitors overexpressing mir-150, lead to significantly reduced mature B cell levels in the circulation, spleen and lymph nodes with little or no alterations in the T cell population [60]. These data demonstrate the implication of these non-coding RNAs on T and B cells differentiation.

Evidence has shown the association between epigenetic mechanisms and the transcriptional control of Foxp3 gene expression, which plays a key role in CD4^+^ T cells differentiation into T_reg_ cells. A motif containing unmethylated CpG, called T_reg_ specific demethylated region (TSDR), was identified on the promoter region of Foxp3, in both developing thymus and mature peripheral T_reg_ cells (CD4^+^CD25^+^Foxp3^+^). Moreover, histone acetylation (H3 and H4 histones) and trimethylation (H3K4me3) was detected in those CD4^+^CD25^+^ T_reg_ cells, while no modifications were shown in non differentiated cells (CD4^+^CD25^−^) [61]. In the same line, it was described a differential methylation pattern on a enhancer sequence and 23 CpG islands present on the Foxp3 promoter region, independent of the presence of TGF-β stimulus. Furthermore, it was demonstrate that CD4^+^CD25^−^ T cells treated with demethylating agent 5-aza-2′deoxycytidine shown an up-regulation of Foxp3 gene expression previously repressed [62]. These data support the idea that epigenetic modifications would directly impact on T_reg_ cells differentiation, being even postulated the DNA methylation status of Foxp3 as a marker of their suppressive potential [63]. MiRNAs also have been associated to differentiation of diverse immune cells types. In T_reg_ cells, the expression of miR-10a is strongly correlated with the expression of Foxp3. Moreover, when the microRNA was suppressed using a specific antagomir, a dose dependent reduction of Foxp3 was achieved [64]. Furthermore, these cells present a loss of functionality when Dicer and others components of the miRnas biogenesis pathway were disrupted [65].

In the differentiation process leading naïve CD4^+^ T cells to Th1 and Th2, trimethylation of lysine 9 on H3 histone (H3K9me3) has been related to transcriptional silencing allowing the differential expression of those genes characteristic for each cell types. Thus, in Th1 cells producing IFN-γ, it was observed a lower percentage of H3K9me3 in the promoter region of the gene encoding this cytokine compared with Th2 cells that produce IL-4. In contrast, these cells showed a lower percentage of H3K9me3 associated with the promoter region of IL-4 than Th1 cells. An inverse effect was described with respect to the acetylation of lysine 9 on H3 with a greater presence in the promoter region of the respective specific expressed gene and consequently associated with transcriptional activation [66].

Epigenetic regulation has also been described by DNA methylation of genes related to CD4^+^ and CD8^+^ memory cells activities. Using naïve CD4^+^ T cells, activated by a transgenic T-cell receptor (TCR), the expression of P-selectin ligands (P-lig) was upregulated, what is characteristic of effectors T cells. Under inflammatory cytokine stimulus (IL-12, IFN-γ and IL-4) the expression of P-lig was detected in a 42% of the cells, which significantly decrease up to 17% in the absence of these stimuli. However, in these conditions, post-treatment of cells with an inhibitor of cytokine methylation increased the percentage of P-lig expressing cells reaching a level similar to the effect shown by cytokine induction, demonstrating that DNA methylation influences the P-lig expression and subsequently the cell differentiation [67]. Similar data were obtained with respect to the expression of CCR in CD4^+^ memory cells, in which methylation-mediated regulation [65].

The influence of epigenetic mechanisms on macrophages has been poorly studied, however, there are some studies providing evidence that as in other cell types, epigenetic modifications are involved in their development. Using cells from mouse bone marrow, it was demonstrated that myeloid cells from hematopoietic progenitor cells have a reduced overall methylation pattern compared to cells that differentiate into lymphoid cells. In addition, after treatment with DNA methyltranferases inhibitors, these progenitor cells had a greater tendency to differentiate into myeloid cells rather than lymphoid cells, demonstrating the importance of DNA methylation on the differentiation process in these cellular types [68]. In the same direction, a drastic demethylation of two cytosine residues located on the CD209 (DC-SIGN) promoter region was detected during the *in vitro* differentiation of monocytes from human blood into dendritic cells (DCs). This finding was correlated with the CD209 gene and protein expression on differentiated DCs cells [69].

Others mechanisms under epigenetic regulation are polarization and activation of macrophages. *Toll-like receptor* (TLR) leads to the activation of various signal transduction pathways (e.g., MAPKs, NFkB, IRF) and inflammatory cytokines (TNF, IL-1β, IL-6, IL-12, p40 y CXCL) that triggers an acute response by M1 macrophages [70]. It has been observed during M1 macrophages differentiation that promoter regions of several genes are characteristically marked by H3K4 trimethylation (H3K4met3) and H3K acetylation [71], as well as by nucleosomes lacking regions located upstream of the transcription start site [72,73,74]. In enhancer sequences of these cells, the presence of H3K4me was associated with chromatin opening and transcriptional permissiveness [75]. In contrast, in the absence of TLR signaling, the transcription process of inflammatory cytokines are controlled by repressors and corepressors, which are able to recruit histone deacetylases and histone demethylases, limiting these markers of transcriptional permissiveness [76]. In addition, it was observed the presence of the repression associated markers H3K9me3, H3K27me3 and H4K20me3 on certain sequences of inflammatory genes [77]. Epigenetic mechanisms have also been described on M2 polarization process. In this line, the Jumonji domain containing-3 (Jmjd3), a histone 3 Lys27 demethylase enzyme, was described as an essential factor in the differentiation process triggered in response to helminth infection [78,79]. Furthermore, in peritoneal macrophages from wildtype and *Jmjd3*^−/−^ mice the expression of Arg1, Ym1, Fizz1 and MR genes, which are characteristically expressed by M2 macrophages, was markedly lower in cells from knockout compared to wildtype mice. Together with these findings, Irf4 was identified as the target gen of Jmjd3, becoming into an important factor involved in the control of gene expression in this macrophage subpopulation [78]. The inhibition of histone deacetylase in RAW 264.7 and primary mouse bone marrow macrophages under LPS stimulus caused a deviation in the distribution of phenotypes from M1 to M2, with repression of CD40, CD80, IL-12 and TNF-a, as well as up regulation of CD86 y IL-10 [80]. Recent reports revealed that miR-125a-3p, miR-26a-2*, miR-181a, miR-204-5p, miR-451 and miR-155 were regulated in M1 polarized phenotype [81,82]. The role of miR-125-3p and miR-155 was confirmed by transfection of mimics, which induced the expression of CXCL9, a characteristic factor expressed during M1 polarization [81]. In the same line, in tumor-associated macrophages (TAMs) the miR-155 role in promoting macrophage polarization from M2 phenotype to M1 phenotype was shown probably by suppressing the C/EBP-β signaling cascade [83]. Furthermore, the definition of M2 phenotype may also be regulated by miRs. MiR-193b was regulated in M2a phenotype while miR-27a*, miR-29b-1*, miR-132* and miR-222* were regulated in M2b phenotype [81]. However, the exact function of these miRs is still unclear. Recently, it was found that miR-let-7c plays an important role in controlling the M2 polarization by targeting C/EBP-β. A higher expression of this miRNA in M2-macrophages was shown compared to M1 phenotype while a decreased expression in M2-macrophages converted to M1-macrophages and a decreased expression in M2-macrophages stimulated with LPS was observed. In addition, knockdown of miR-let-7c in M2 phenotype stimulated M1 polarization and diminished M2 phenotype [84]. By contrast, peritoneal macrophages extracted from Akt2^−/−^ mice exhibited an M2 macrophage phenotype probably due to higher levels of C/EBP-β. This was correlated with reduced levels of miR-155 and increased expression of Arg1, a well-known M2 marker [85].

## 4. Regulation of Epigenetic Mechanisms by Polyphenols

Several studies have shown that polyphenols are able to modulate epigenetic mechanism including either DNA methylation or histone modifications. In this regard, a number of natural compounds have been identified as histone deacetylase (HDAC) inhibitors (EGCG, curcumin, genistein, quercetin), histone acetyltranferase (HAT) activators (genistein), HAT inhibitors (EGCG, curcumin), silent information regulator (SIRT) activator (resveratrol) or SIRT inhibitor (genistein) [86].

The modulation of epigenetic mechanisms by polyphenols has been reported showing an inhibitory effect of EGCG on DNA methyltranferase-1 (DNMT1) together with transcriptional reactivation of suppressed genes. This inhibitory effect could be determined by a direct interaction between EGCG and DNMT1 in according to *in silico* molecular modeling studies [87]. Later studies described two mechanisms of DNMT1 regulation. Catechol-containing polyphenols showed an inhibitory effect by S-adenosylhomocysteine (SAH) production derived from its own methylation process, using S-adenosylmethionine (SAM) as methyl donor [71,72]. This process promotes SAH accumulation, acting as noncompetitive inhibitor of DNMTs. Moreover, EGCG showed a direct inhibitory effect on DNMT1, mediated probably by the interaction described above [88]. Curcumin showed a similar inhibiting effect probably by a covalent interaction [89]. In relation to the histone modulation by polyphenols, it was shown that EGCG is able to induce re-expression of the silenced tumor suppressor genes, p16INK4a and Cip1/p21, by partial inhibition of HDAC activity and increased acetylation of lysines 9 and 14 on H3 histone (H3-K9 and 14) and acetylated lysine 5, 12 and 16 on H4 histone besides to decrease the levels of methylated H3-Lys 9 [90]. Moreover, curcumin inhibited HAT activity by inducing proteasome-dependent degradation of p300 in cancer cells [91] besides to inhibit the expression of p300, HDAC1, HDAC3, and HDAC8 proteins in Raji cells, modulating the NFκB signaling pathway [92]. Furthermore, quercetin induced HAT activation and HDAC inhibition in HL60 leukemia cells promoting increased histone H3 acetylation and inducing FasL-related apoptosis [93]. Genistein activated tumor suppressor genes by demethylation and acetylation of H3-K9 at the PTEN and the CYLD and decreasing endogenous SIRT1 activity, promoting acetylation of H3-K9 at the p53 and the FOXO3a promoter [94].

Furthermore, the epigenetic modulation by polyphenols also affects the expression of miRNAs participating in many biological processes in several cellular types. This regulatory effect has been observed in the hepatic HepG2 cell line in which the treatment with EGCG shown a decrease on miR miR-30b*, miR-453, miR-520e, miR-629, and miR-608 [95]. By using an *in vivo* model to evaluate the effect of dietary supplementation with several polyphenols on the miRNAs profile expression in hepatocytes, a modulatory effect was observed for five miRNAs commonly affected by the tested polyphenols [96]. These data demonstrate the ability of polyphenols to modulate the gene expression through the regulation of epigenetic mechanisms (Table 1) and creates an interesting target of study, aiming to clarify the mechanisms by which the polyphenols modulate microRNAs and, thus, their target mRNAs, leading to gene expression restraining.

**Table 1:** Epigenetic mechanisms regulated by polyphenols.

Polyphenols	Associated epigenetic mechanism	Transcriptional effect	References
Epigallocathechin-3-gallate	DNMT1 inhibition	Expression	[87]
	HDAC inhibition	Expression	[90]
	miRNAs repression	Expression	[95]
Curcumin	HAT inhibition	Repression	[91]
Quercetin	HAT activation and HDAC inhibition	Expression	[93]
Genistein	Histone demethylation, HAT activation and SIRT inhibition	Expression	[94]

## 5. Modulation of Immune Function by Polyphenols through Epigenetic Mechanisms

**Figure 3:**
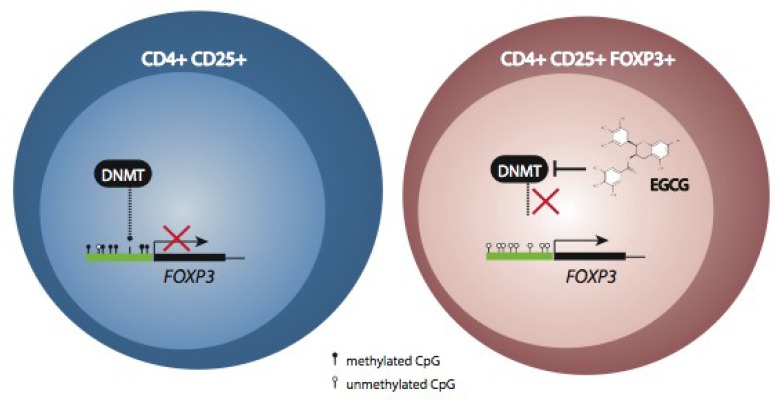
Immuno-modulation through epigenetic mechanism regulated by polyphenols.

The findings presented here shown that polyphenols from different sources are capable to regulate the immune function. Also, such regulation can be determined by modulation of gene expression of factors that plays key roles in activation and differentiation of cell types involved in immune function by well known epigenetic modifications. Moreover, these epigenetic modifications can be regulated by polyphenols, allowing hypothesize that polyphenols-modulated epigenetic modifications are involved in the regulation of immune response by these bioactive compounds (Figure 3). Although few studies focused on this idea, recent data support this hypothesis. *In vitro* assays using Jurkat T cells showed a significant increase of Foxp3 and IL-10 expression after treatment with EGCG and green tea extract containing an equivalent concentration of EGCG [20]. Furthermore, the same effect was observed in cells under treatment with demethylating agents, indicating that the variation induced on the methylation pattern of these cells plays an important role in the transcriptional reactivation of the previously suppressed genes. Together with these findings, it a decrease of the global DNA methylation and the expression of three DNMTs were described in the Jurkat T cells. The same study described a significant increase in T_reg_ frequencies in the spleens, pancreatic lymph nodes, and mesenteric lymph nodes of mice treated with EGCG and these cells were functionally active, inducing suppression of activation and differentiation of T cells [20]. High levels of glucose can promote a pro-inflammatory state in human monocyte by acetylation of NF-κB and subsequent cytokines gene expression. In this regard, fisetin treatment inhibited the expression of NFκB target genes, including IL-6 and TNF-α in THP-1 monocytes cells exposed to high-glucose concentrations (HG-cells) [97]. This flavonol inhibited the p65 acetylation, causing inhibition of the NFκB transcription activity. In addition, fisetin could inhibit inflammation by up-regulation of HDAC activity and inhibition of HAT activity in HG-cells, preventing NF-κB-mediated chromatin acetylation and subsequent transcription of cytokines [97]. Similarly, also in human THP-1 monocytes exposed to hyperglycemic conditions, curcumin inhibited cytokines release and NF-κB transactivation. In addition, HAT activity, as well as the levels of p300 and CBP/p300 acetylation, was reduced while HDAC2 expression was induced. Since p300 histone acetyltransferase is a coactivator of NF-κB, curcumin decreases HG-induced cytokine release in monocytes via epigenetic changes involving NF-κB [98]. 

Finally, *in vitro* and *in vivo* assays conducted to evaluate the anti-inflammatory effect of quercetin and its metabolites showed a decreased in TNF-α, IL-6, IL-1β, macrophage inflammatory protein 1α (MIP-1α) and iNOS mRNA. Moreover, it was observed an increase on heme oxigenase 1 protein, known as chorin inflammatory antagonist. These findings were accompanied with lower expression of proinflammatory miR-155, suggesting an important role in the anti-inflammatory effect of quercetin [99].

## 6. Conclusions

These data indicate that polyphenols are able to modify epigenetic mechanisms promoting immune modulation. Actually, the effects of polyphenols on epigenetic mechanism are yet poorly described and represent an interesting field of study. As epigenetic mechanisms are involved in the control of gene expression, thus, acting on the maintenance of functionality of numerous physiological processes, the modulation of epigenetic modifications by polyphenols is of great interest in immune-mediated diseases. 
